# Melatonin/Cyclodextrin Inclusion Complexes: A Review

**DOI:** 10.3390/molecules27020445

**Published:** 2022-01-10

**Authors:** Aikaterini Sakellaropoulou, Angeliki Siamidi, Marilena Vlachou

**Affiliations:** 1 Section of Pharmaceutical Chemistry, Department of Pharmacy, School of Health Sciences, National and Kapodistrian University of Athens, Panepistimioupoli-Zografou, 15784 Athens, Greece; aiksakell@pharm.uoa.gr; 2 Section of Pharmaceutical Technology, Department of Pharmacy, School of Health Sciences, National and Kapodistrian University of Athens, 15784 Athens, Greece; asiamidi@pharm.uoa.gr

**Keywords:** melatonin, cyclodextrins, inclusion complexes

## Abstract

Melatonin (MLT) is involved in many functions of the human body, mainly in sleeping-related disorders. It also has anti-oxidant potential and has been proven very effective in the treatment of seasonal affective disorders (SAD), which afflict some people during short winter days. Melatonin has been implicated in a range of other conditions, including Parkinson’s disease, Alzheimer’s and other neurological conditions, and in certain cancers. Its poor solubility in water leads to an insufficient absorption that led scientists to investigate MLT inclusion in cyclodextrins (CDs), as inclusion of drugs in CDs is a way of increasing the solubility of many lipophilic moieties with poor water solubility. The aim of this review is to gather all the key findings on MLT/CD complexes. The literature appraisal concluded that MLT inclusion leads to a 1:1 complex with the majority of CDs and increases the solubility of the hormone. The interactions of MLT with CDs can be studied by a variety of techniques, such as NMR, FT-IR, XRD and DCS. More importantly, the in vivo experiments showed an increase in the uptake of MLT when included in a CD.

## 1. Introduction

Cyclodextrins (CDs) are small carbohydrates involved in a variety of purposes including medicinal applications. The first report on CDs appeared in 1891 by Villers and since then, the research around the topic has proliferated [1]. In 1948 scientists, after purification, identified that CDs could consist of six, seven or eight *α*-d-glucopyranose units in the 4-C1 chair conformation linked with an *α*-1,4 bond, thus giving *α*-CD, *β*-CD and *γ*-CD, respectively [2,3]. These glycose units form a cyclic structure often referred to as a ring, doughnut, cylinder or truncated cone that can host molecules. CDs are widely produced by starch with the usage of cyclomaltodextrin glucanotransferase, an enzyme that naturally occurs in *Bacillus macerans* [1]. The secondary hydroxyls of the glucose units are located on the wider edge, while the primary hydroxyls are on the narrower of the exterior of the cone. The interior consists of the carbon and ether type oxygen chains [1]. The hydrophilic exteriors as well as the lipophilic interiors are the characteristics that make these molecules so important for the pharmaceutical field. However, the naturally occurring *β*-CD has limited water solubility compared to *α*- and *γ*-CD. To circumvent this problem, several modifications were made in order to break the intramolecular hydrogen bond grid, and today a great variety of substituted CDs are used, such as the 2-hydroxypropyl derivatives (HP-*β*-CD), the sulfobutylether-*β*-CD (SBE-*β*-CD) and randomly methylated *β*-CDs (RAMEB or RM-*β*-CD) [4].

CDs have been used as cages in order to host molecules to enhance the solubility of lipophilic drugs, improve their stability by limiting dehydration, hydrolysis, oxidation and photodecomposition, and increase their bioavailability. In addition, CDs can be used in order to reduce irritation from the direct contact of the drug with biological membranes and also drug toxicity by minimizing the effective dose and by limiting the crystallization of poorly soluble drugs in parenteral route administration [5]. Regarding the solubility of drugs, CDs can be used to increase their dissolution kinetics, leading to higher bioavailability, mainly on those that are characterized as Class II drugs in the Biopharmaceutical Classification System (BCS) [6,7]. In addition to the effect on drug solubility and dissolution, CDs affect drug permeability through biological barriers where a lipophilic membrane is under an aqueous exterior. CDs help by solubilizing the drug in the aqueous phase and by that, more molecules reach the biological membrane, increasing its bioavailability. However, excessive amounts of CD may inhibit the absorption of the drug in the gastrointestinal (GI) tract. The bonds formed at the drug/CD complex are non-covalent, and thus, the entrapped drug molecules are in equilibrium with the free drug [7]. CDs have been used in different kinds of oral drug delivery systems depending on the need for immediate, prolonged, modified or delayed release [5].

The pineal hormone melatonin (MLT) plays a major role in sleep regulation. In 1958, Lerner isolated MLT from bovine pineal glands, and in 1959 its structure was elucidated as *N*-acetyl-5-methoxytryptamine [8,9]. MLT’s biosynthesis takes place at night, when there is no presence of light. It is mainly produced in the pineal gland; however, studies have shown that traces can be found in the GI tract, the leukocytes and the retina. The biosynthesis starts from tryptamine and includes the formation of serotonin. Serotonin is then *N*-acetylated and *O*-methylated in order to give MLT. The metabolism of MLT occurs mainly in the liver; however, studies have shown that MLT is also metabolized in the brain [10] and the retina [11]. MLT has a variety of actions, including the ability to regulate the circadian rhythm in mammals. This action is mediated by the activation of MLT receptors. The receptors are categorized as high affinity (MT1 and MT2) and low affinity receptors (MT3) [12]. There are also studies that indicate the existence of MLT nuclear receptors RZR/ROR*α* and RZR*β* [10]. MLT has been used for its benefits in treating sleeping disorders such as difficulty in falling and staying asleep, jet-lag, sleeping difficulty in blind people, circadian rhythm sleep disorders (CRSD) and delayed sleep phase syndrome (DSPS) [13,14]. By activating MT1 receptors at the suprachiasmatic nucleus (SCN), MLT inhibits the neuronal firing rate while activating MT2; the hormone synchronizes the phases of the circadian rhythms generated within the SCN, thus playing an important role in controlling sleeping [15]. Besides its effect on sleep, MLT also shows noteworthy antioxidant activity by reacting with free radicals as well as enhancing the endogenous antioxidative enzymes [16]. MLT has anti-inflammatory activity [17], and it could be beneficial in a variety of diseases, such as hemorrhagic shock, cancer [18], and autoimmune [19], cardiovascular [16] and neurodegenerative diseases [20,21]. Moreover, it shows osteoinductive and anticarcinogenic properties in different osteosarcoma cell lines, and due to its strong antioxidant activity, MLT can be used for the treatment of granular dystrophy type 2.

Due to the ability of MLT to regulate sleep, it is widely administered as a supplement or as a prescription drug with a variety of routes of administration being available. So, MLΤ can be given orally in forms for immediate or sustained release as well as via transdermal, intranasal or transmucosal routes [22]. As a drug moiety, it is classified as a Class II drug in the BCS and has limited solubility in water (0.1 mg/mL) with a logP = 1.6 [23,24]. This limitation may possibly lead to problems with absorption and thus the bioavailability of these formulations, resulting in a variation in MLT levels in the serum of patients [25,26]. Hence, it is critical to find a way to improve its bioavailability in order to achieve better results after administration. To overcome these limitations, researchers have prepared MLT/CD complexes and studied their properties. The aim of this review is the compilation of key up-to-date findings on MLT/CDs complexes.

## 2. Results

In the present manuscript, 21 research articles were reviewed and analyzed in order to draw conclusions on the purpose of MLT inclusion in CDs and the methods used to achieve it. A summary table, Table 1, was constructed, and the studies included were mainly grouped by dosage form and drug release. The release pattern was reported, as indicated by the author(s) in each publication. Additionally, the CDs used, the methods of inclusion, and the experiments conducted were reported, along with the pH of the solvents used in the respective experiments.

## 3. Discussion

**Figure 1:**
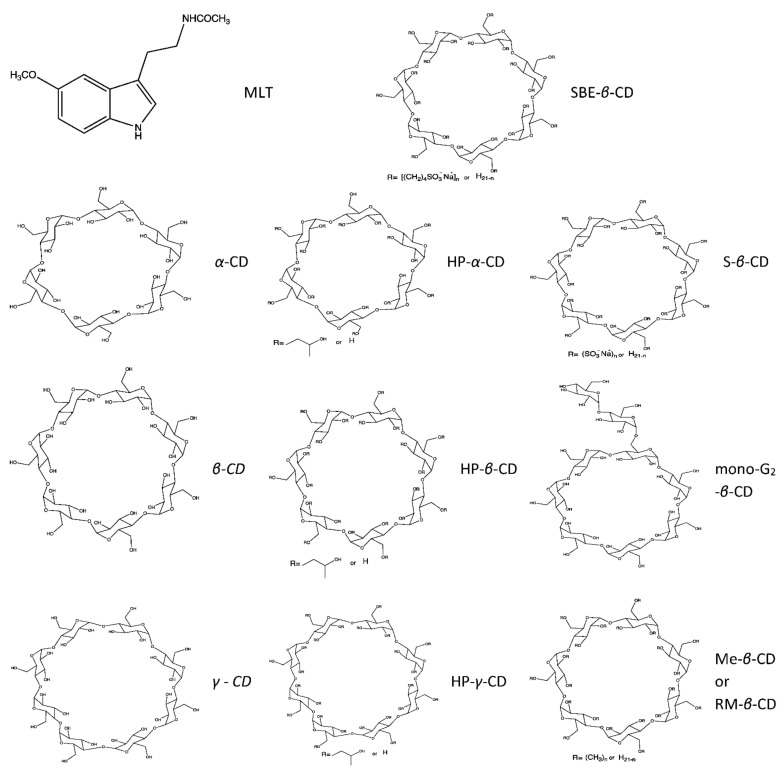
Structures—MLT: melatonin, *α*-CD: *α*-Cyclodextrin, *β*-CD: *β*-Cyclodextrin, *γ*-CD: *γ*-Cyclodextrin, HP-*α*-CD: Hydroxypropyl-*α*-CD, HP-*β*-CD: Hydroxypropyl-*β*-CD, HP-*γ*-CD: Hydroxypropyl-*γ*-CD, SBE-*β*-CD: Sulfobutylether-*β*-CD, S-*β*-CD: Sulfated-*β*-CD, mono-G2-*β*-CD: Mono-6-*O*-*α*-maltosyl-*β*-CD, Me-*β*-CD: Methyl-*β*-CD, RM-*β*-CD: Randomly methylated-*β*-CD.

MLT/CD inclusion complexes (in solid, hydrogel and liquid form) have been prepared by a variety of methods (physical mixing, kneading, freeze-drying, etc.) in order to improve MLT’s hydrophilicity and to study their properties (solubility, stability, drug release, etc.). The most commonly used CDs for MLT inclusion are *β*-CD and HP-*β*-CD, which offer enhanced results. As stated previously, the medium size *β*-CD cavity allows for deep MLT penetration [44]. Further, modified CDs and particularly HP-*β*-CD can act as penetration enhancers by solubilizing MLT [37]. The structures of MLT and all reported CDs are presented in Figure 1.

The *β*-CD cavity has a diameter of 0.60–0.65 nm and a height of 0.78 nm [48]. Its medium-size cavity is ideal for the encapsulation of drugs and other small molecules, as it allows deep penetration into the cavity and the development of appropriate interactions between the CD and the molecule. The oxygen atom of the methoxy group possibly forms a hydrogen bond with the axial C-3 and/or C-5 proton of the CD, while, H-4, H-7 and H-6 form hydrogen bonds with the anomeric oxygens of the inner part of the CD cavity and H-2 forms a hydrogen bond with the C-3 or the C-5 OH. Probably only the indolic part of MLT enters the cavity, forming hydrophobic interactions with the CD carbons and the aromatic protons, as well as the methyl part of the methoxy group, which is also involved via hydrogen bonds or other interactions in the stabilization of MLT into the CD cavity [34].

### 3.1. Solid Dosage Forms

Topal et al. [27], in an effort to improve MLT’s hydrophilic properties, prepared inclusion HP-*β*-CD/MLT complexes using microwave. The complexes were then loaded into chitosan scaffolds and studied in vitro for their effect on osteosarcoma cells (MG-63). The method of preparation included the addition of the MLT and the CD in a 50% *v*/*v* water/ethanol solution, using 3 different microwave powers (150, 600 and 900 W), for 90 s at 60 °C, with the optimal power being 900 W, where MLT showed the highest solubility (0.043 M). In these conditions, the complexes had the highest *K*s (62.16 M^−1^) and thus the highest stability, according to the phase solubility studies conducted. After the inclusion, the complexes were dried under vacuum and obtained as white solids, which were analyzed with Fourier transform infra-red (FT-IR), differential scanning calorimetry (DSC), X-ray diffraction (XRD) and nuclear magnetic resonance (^1^H-NMR, ^13^C-NMR). These complexes were then loaded into chitosan scaffolds followed by in vitro release kinetics determination in a phosphate buffered saline (PBS) (pH = 7.4) solution containing 0.1% (*w*/*v*) sodium azide. These studies indicated that 84% of MLT was released from the scaffold that was loaded with the MLT/CD complex, compared to 71% released from the scaffold loaded only with MLT. This differentiation arises because when loading the scaffold, the amount of the inclusion complex loaded is 2-fold higher than the amount of MLT loaded alone. Thus, because of the higher difference in concentration, more MLT is released. From the ^1^H-NMR studies, it can be deduced that MLT incorporates to the wide side of the CD cavity, near the H-3 of the HP-*β*-CD, leading to a higher chemical shift than the H-5. From the chemical shifts of ^13^C-NMR, the upfield resonating carbons are those that interact with the CD cavity, whilst the downfield appearing carbons are those that are externally close to the wide part of the CD cavity; this might show that the aromatic part of MLT is incorporated into the CD, while the aliphatic part interacts with the wide side of the cavity [27]. As a continuation of this work [27], Cetin et al. prepared a chitosan/hydroxyapatite (chitosan/HAp) scaffold, which was then loaded with the MLT/HP-*β*-CD complex [28]. For the preparation of the scaffolds in a 2% (*w*/*v*) chitosan solution in distilled water that contained 0.2 M acetic acid, 1.5% (*w*/*v*) HAp particles in bead were added, and the solution after mixing was lyophilized in a freeze-dryer. After lyophilization, the scaffolds were treated with sodium carbonate (1 M), washed with distilled water and dried in the freeze-dryer. For the inclusion of MLT in HP-*β*-CD, the method that has been described by Topal in 2015 [27] was followed. In order to embed the complex into the chitosan/Hap scaffold, the scaffolds were added to a solution of MLT/HP-*β*-CD in water and refrigerated overnight. The scaffolds were then removed from the solution, frozen and lyophilized, and then characterized by scanning electron microscopy (SEM) and attenuated total reflectance Fourier transform infra-red (ATR-FT-IR). The in vitro MLT release studies, conducted in phosphoric buffer (pH = 7.4) containing 0.1% (*w*/*v*) sodium azide, showed a rapid release of 80% of MLT in the first 20 min, with the remaining quantity being released over a period of 5 h. The chitosan/HAp loaded scaffolds inhibit the proliferation of MG-63 cells in the G_0_/G_1_ phase, with the pattern of immediate and short-time release of MLT being critical for this inhibition [28]. 

A group of scientists prepared MLT and doxorubicin (DOX) co-delivery systems via a functionalized graphene dendrimeric system. The synthesis of the dendrimeric system involves two parts. The first part refers to the formation of the amine-functionalized *β*-CD (AF-*β*-CD), while the second includes the formation of the epoxy-functionalized graphene oxide. The two parts are finally combined to create the dendrimeric system. The synthesis of AF-*β*-CD starts with the formation of tosylate *β*-CD (Ts-CD), by the reaction of *β*-CD with *p*-toluenesulfonylchloride. The Ts-CD is then reacted with ethylenediamine (EDA) to form ethylenediamine-*β*-CD (EDA-CD) and then with methyl acrylate (MA) to give methyl acrylate-ethylenediamine-*β*-CD (MA-EDA-CD), which was reacted with EDA to form AF-*β*-CD. The synthesis of the epoxy-functionalized graphene includes two steps. First, by reacting graphite with NaNO_3_, H_2_SO_4_ and KMnO_4_, the graphene oxide (GO) is formed. In a water suspension of the GO, epichlorohydrin (ECH) was added to give the epoxy-functionalized GO. Lastly, the AF-*β*-CD was added in a dispersion of the GO-epoxy in phosphate buffer, followed by adjustment of the pH at 10.5 to give AF-*β*-CD/GO, which was then reacted with FeCl_3_x6H_2_O and FeCl_2_x4H_2_O to give the amine-functionalized *β*-cyclodextrin-grafted graphene oxide/Fe_3_O_4_ nanoparticles (AF-*β*-CD/GO/MNPs). The carrier was then suspended in PBS, DOX was added, and the mixture was stirred at room temperature. The solid was freeze-dried and likewise was loaded with MLT and freeze-dried once again to give the final system. MLT was released from the nanocarrier in a sustained pattern in the first 6 h (65.69% of the overall MLT), reaching 88.96% at t = 12 h and pH = 5.3. In the studies conducted at pH = 7.4, MLT was not detectable [29].

Another study focused on the preparation and characterization of MLT complexes with randomly methylated *β*-CD (RM-*β*-CD). Two complexes with different ratios of MLT and CD were prepared by the co-evaporation method. The MLT and the CD were dissolved, in both cases, in isopropanol. The solutions were then shaken, and the alcohol was evaporated in vacuo at 55 °C to afford the complexes as white powders. The solubility tests revealed that 0.08 M RM-*β*-CD increased MLT solubility by 8.5-fold. According to the phase solubility studies, the RM-*β*-CD/MLT complex has a stability constant *K*s = 272.0 M^−1^, which indicates the compatibility of MLT with the CD and the formation of a stable complex. From the stability studies, it was found that by increasing the temperature, the *K*s and thus the stability of the complex decreases, due to the fact that at high temperatures the hydrogen bonds are weakened, leading to an insufficient interaction between the drug and the host. The negative ΔH for the inclusion complex points to strong van der Waals–London interactions. From the thermodynamic measurements, a negative ΔG_0_ indicates that the formation of the complex is spontaneous and favorable, while the negative ΔH_0_ indicates that it is an exothermic reaction, due to the formation of hydrogen bonds and hydrophobic reactions. From the thermograms, it can be assumed that MLT forms weak interactions with the CD’s cavity in the presence of unbound MLT molecules in the solution. For the complex where CD is at a higher concentration, the formation of the complex is successful; hence, the ratio of MLT/CD must be 1:2 to ensure full inclusion. Finally, regarding the stability of MLT, the encapsulated MLT seems to have less photosensitivity than the free MLT [30].

A group of researchers investigated in detail the physical properties of inclusion complexes of MLT with a variety of modified CDs (*α*-CD, *β*-CD, *γ*-CD, HP-*α*-CD, HP-*β*-CD, HP-*γ*-CD, mono-6-*O*-*α*-maltosyl-*β*-CD (mono-G_2_-*β*-CD), methyl-*β*-CD (Me-*β*-CD), SBE-*β*-CD). Amongst all these complexes, the highest complexation ability was observed with SBE-*β*-CD. This complex increased the solubility of MLT by 11-fold, at pH = 7.5, and also showed the best stability (highest *K* value) in the phase solubility studies, in comparison to the other complexes studied. Probably, this is due to the anionic group of the particular cyclodextrin, which in the complex system interacts in an electrostatic fashion with the molecule of melatonin at pH 7.4 and not at pH 1.2, where its sulfated groups are protonated. The stability of the SBE-*β*-CD/MLT complex increased with an increase in pH, with the *K* value at pH = 11 being 520 ± 30 M^−1^. Spectrofluorimetry at 335 nm of the CD/MLT complexes, at pH = 7.5, confirmed that the SBE-*β*-CD had the highest *K* and was the most stable. The solid systems of MLT and SBE-*β*-CD were prepared by the physical mixing, kneading and freeze-drying methods. In the physical mixing, MLT and CD in a 1:1 molar ratio were mixed, while in the kneading method, MLT and CD of the same ratio were mixed in the presence of a small amount of ethanol. For the freeze-drying method, the MLT and CD were dissolved in water with a very small amount of sodium hydroxide solution (6 M), followed by concentration in vacuo at −40 °C. NMR, XRD and DSC studies suggested that with the physical mixing and kneading methods, MLT kept its crystalline structure, while with the freeze-drying method, the solid was amorphous, and thus, MLT was fully incorporated into the CD cavity. In the ^1^H-^1^H ROESY NMR experiment, a correlation was shown between the H-3 of the SBE-*β*-CD (3.71 ppm) with the H-6 (6.79 ppm) and the H-4 (7.01 ppm) of MLT. No cross peaks between the MLT alkyl group (3.33 ppm) and the H-3 of the SBE-*β*-CD were observed, but there was an interaction between the alkyl group and the sulfobutylether groups (2.80 ppm). All of these findings lead to the conclusion that the 5-methoxyphenyl group is surrounded by the cavity of the CD and the alkyl chain interacts with the sulfobutylether chain of the CD [31].

In another research work, the formation and stability of inclusion complexes of MLT with natural CDs in the solid phase were also investigated. FT-IR and XRD studies were conducted on α-CD, *β*-CD and *γ*-CD physical mixtures and complexes with MLT. The complexes of MLT/CD used were either lyophilized or used as crystalline complexes. The lyophilized complexes were prepared by mixing a MLT solution (0.002 M) with the appropriate CD, ultrasonic treatment, freezing and freeze-drying, whereas the crystalline complexes were prepared similarly to the lyophilized, but dried under vacuum instead of being frozen and freeze-dried. From the FT-IR experiments, it was clear that the physical mixtures of CDs do form MLT/CD complexes, but there are still high concentrations of crystalline MLT and CDs, and thus, the peaks of free MLT still exist without substantial variation. On the other hand, on the spectra of complexes, we could see convolutions and shifting of important MLT peaks, especially on the polar groups, as well as the disappearance of some MLT peaks. Both inspections imply the formation of the complex and the formation of intramolecular stabilizing bonds. From the FT-IR spectra, it can be implied that the amidic carbonyl (CO) forms a hydrogen bond with the OH of the exterior of the CD outer ring along with non-polar interactions. In the X-ray analysis, the MLT and CD physical mixture and the MLT/CD complex were studied. For the preparation of these complexes, MLT and the CD used were in a 1:1 and a 2:1 molar ratio. Similarly to the FT-IR experiment, X-ray analysis showed that in the physical mixture, there was a large amount of unbound MLT, whereas on the 1:1 MLT/CD colmplex this amount was smaller and for the 2:1 ratio negligible [32]. 

Bilayer tablets were also prepared for the modified release of MLT. In particular, the tablet consisted of two parts, a fast-release and a slow-release fraction. In the fast-release layer of this bi-layered tablet, *β*-CD was used to improve MLT’s solubility, whereas hydroxypropylmethylcellulose (HMPC) and Carbopol 971 P matrix (CP-971P) were used to achieve a sustained release of the hormone. The complexes were prepared either by the solid dispersion method, where MLT and the CD were dissolved in ethanol (50%) and the solvent was then evaporated, or by the kneading method with the use of a small quantity of water. From the solubility tests, it was clear that the solubility increased linearly as the CD concentration increased. The stability constant for the complex was calculated from the phase solubility diagrams and was found to be 50.11 M^−1^. The complexes were characterized by DSC, FTIR and SEM, all of which concluded that in the kneading method there was a better complex formation. The amount of MLT released from the tablets containing the MLT/*β*-CD complex made with the kneading method was 84% in 30 min, while the tablets that contained the complex formed by the solid dispersion method and the tablets containing unbound MLT released the hormone at percentages 66 and 51, respectively, in 30 min [33]. 

Vlachou et al. prepared a series of controlled-release tablets that contained MLT inclusion complexes in a variety of CDs and investigated MLT’s release from the tablets at pH = 1.2 and 7.4 [34]. The CDs were used either as excipients or as molecules to host the guest hormone. All of the formulations included MLT, a specific cyclodextrin, or the complex of MLT with the CD, HMPC, Avicel, sodium alginate and magnesium stearate to form tablets of 200 mg total weight. The inclusion of MLT in the CD cavity was effected by the co-evaporation method. MLT and the appropriate CD were diluted in water and stirred, and the solvent was evaporated under vacuum. The *K* values were calculated from the Benesi–Hildebrand equations and were found between 1006.9–5431.86 M^−1^ for the complexes with α-CD, *β*-CD, sulfated-*β*-CD (S-*β*-CD), *γ*-CD, HP-*α*-CD and HP-*γ*-CD. For the complex of MLT with HP-*β*-CD, the *K* value obtained was 24,092.80 M^−1^. This rise is probably caused by the formation of additional hydrogen bonds with the additional OHs of the CD’s exterior. From the ^1^H-NMR spectrum of MLT/*α*-CD, the downfield shift of H-7 and H-4 indicates that these protons form hydrogen bonds with the C-3 and C-5 OHs of the *a*-CD. MLT probably enters the cavity from the benzene site of the indole nucleus, and its orientation differentiates when entering the HP-α-CD cavity. The data from the ^1^H-NMR of the MLT/*β*-CD complex show a shift in all of the *δ* values of MLT aromatic protons with the H-2 proton possibly forming a hydrogen bond with the OH of C-3 or C-5. The chemical shifts of the protons of the side chain of MLT were not affected, meaning that probably only the indolic part of MLT enters the cavity. From the ^13^C NMR spectra, it becomes apparent that the aromatic protons corresponding to the aromatic carbons as well as the methyl part of the methoxy group were involved via hydrogen bonds or other interactions in the stabilization of MLT into the CD cavity. The oxygen atom of the methoxy group possibly forms a hydrogen bond with the axial C-3 and/or C-5 proton of the CD, while H-4, H-7 and H-6 form hydrogen bonds with the anomeric oxygens. From the ^1^H-NMR studies on the MLT/*γ*-CD complex, it becomes apparent that the interactions developed between the hormone and CD are weak attractive forces and not hydrogen bonds. The dissolution profile of these tablets was studied at pH = 1.2 and 7.4. The dissolution rate and thus MLT’s release from all of the tablets that contained complexes of MLT/CD, and particularly those that used *β*-CD (natural *β*-CD or HP-*β*-CD), were enhanced compared to that from the tablets that contained only CD as an excipient. The *β*-CD complex released 100% of the active substance in 3 h at 1.2 pH, while it needed 8 h to release 90% at pH 7.4. For the MLT/HP-*β*-CD complex, the release reached 100% at both pHs, taking 5 h at 1.2 pH and 1.5 h at pH 7.4. The MLT/β-CD and the MLT/HP-β-CD complexes seem to form stronger host–guest interactions at pH 1.2, as compared to pH 7.4. This reflects the relative % release of melatonin at these pH values [34].

Apart from solid scaffolds and tablets, researchers have also prepared and studied MLT loaded *β*-CD nanosponges (NSs). The NSs have several advantages in comparison with other drug delivery systems, and recently they have been established for targeted drug delivery. Besides targeted delivery, NSs are non-toxic and can encapsulate a great variety of substances and reduce their potential side effects. In addition, the formulations of drugs with NSs are stable over a wide range of pH and temperature, improving the stability of chemically unstable substances [49]. *β*-CD was dissolved in *N*,*N*-dimethylformamide (DMF), carbonyldiimidazole (CDI) was added (*β*-CD/CDI ratio 1:8), and the resulting mixture was heated. The obtained monolith was crumbled in a mortar and purified via Soxhlet extraction in ethanol and dried to give a white powder. In order to load MLT into the complex, the hormone and the CDI-NS (1:5 ratio) were dissolved into a 4:6 water/ethanol mixture. The suspension was stirred at room temperature, filtered under vacuum, rinsed with deionized water, filtered and then lyophilized. From the characterization of the complex, it is clear that the CDI-NSs have the tendency to aggregate, while the loaded MLT was about 8% and none of its peaks were visible in the XRD spectra. Regarding release kinetics, in vitro studies were conducted in Franz diffusion cells, where the receptor chamber was filled with phosphate buffer (pH = 7.4) and the donor’s chamber with the cotton fabric with MLT. The MLT/CDI-NS complex showed zero-order kinetics, with the release being a reservoir diffusion-controlled system regardless of the MLT concentration [35]. 

### 3.2. Semisolid Dosage Forms

Semisolids were also investigated in a study that analyzed the formation of a series of hydrogels, synthesized by the copolymerization of a monovinyl CD monomer with 2-hydroxyethyl acrylate (HEA). In addition, the controlled drug release of MLT from these hydrogels was also examined. The first step, in order to form the hydrogel, was the synthesis of a monovinyl-*β*-CD monomer (GMA-EDA-*β*-CD). Mono-6-*O*-Ts-*β*-CD was reacted with excess EDA to form EDA-*β*-CD, which was then reacted with GMA to form GMA-EDA-*β*-CD as a white solid. This monomer was then reacted with HEA in the presence of an ammonium persulfate/sodium bisulfate redox system acting as an initiator for the copolymerization. To a mixture of water/methanol (75/25) and 0.9% MLT, the hydrogels in the form of discs were added and left to swell in the solution at 25 °C in order for MLT to load. Then, the loaded discs were left to dry at ambient conditions and in a vacuum oven at 30 °C. The hydrogels have relative stability in a pH range from 1.4 to 7.4. The release from the hydrogels is correlated with the amount of *β*-CD in the hydrogel. Hydrogels with higher amounts of *β*-CD tend to lead to a lower MLT release rate, resulting in a sustained release [36].

### 3.3. Liquid Dosage Forms

Furthermore, scientists were also engaged with liquid forms of MLT/CD complexes. In particular, inclusion complexes of MLT with HP-*β*-CD and RM-*β*-CD were prepared by the flash evaporation method. MLT and the CD were dissolved in isopropanol to obtain a 1:1 MLT/CD molar ratio solution. The solvent was then evaporated under reduced pressure, at 55 °C, and the white powder obtained was further dried and stored in a desiccator and used for characterization. The RM-*β*-CD/MLT complex was more stable, possibly due to spatial compatibility of MLT and the strength of the occurring interactions, with the calculated stability constants from phase solubility studies being 193.6 M^−1^ and 263.9 M^−1^ for the HP-*β*-CD/MLT and RM-*β*-CD/MLT complexes, respectively. The nasal formulation was prepared by adding the microionized MLT to a dispersion of HMPC in a Tween 80 solution (0.1% in purified water) and placing in a shaker. From the phase solubility tests, it is evident that both CDs that were used increased MLT’s solubility, with 10% HP-*β*-CD being able to solubilize the entire 1% MLT suspension and the RM-*β*-CD being an even better solubilizer. However, CD has a strong affinity for MLT, thus behaving as a weak penetration enhancer in higher concentrations. In the concentration of 1%, both CDs were found to improve the nasal absorption of MLT [37]. In addition, complexes of MLT with *α-*, *β-*, *γ-* and HP-*β*-CD were prepared to be used as an eye solution formulation. The complexes were further tested for their modification of the solubility of MLT and their ability to be used for the intracorneal delivery of the hormone. For the solubility study, MLT was added to water solutions of increasing CD concentrations and centrifuged, and the supernatant was diluted and determined by HPLC-UV at 223 nm. From the solubility studies, it appeared that the HP-*β*-CD had the most promising results (MLT solubility from the complex was 2.75 mg/mL), and therefore, it was used for the intracorneal delivery system. The MLT/HP-*β*-CD complex was prepared by dissolving MLT into PBS (pH = 7.4) and the CD into PBS, thus creating the stock solutions. Afterwards, the two stock solutions were mixed in different quantities in order to create four different MLT/CD complex solutions. From these solutions the one containing MLT 0.05% and HP-*β*-CD 0.3% showed less irritation to rabbit conjunctiva. For aqueous solutions such as eye drops, the CD acts as a solubilizer, keeping the molecules of MLT in solution and helping them come into contact with the mucin layer of the eye and thus increasing the permeability of the hormone. However, too much CD can cause the opposite effect by inhibiting the permeation, due to the fact that the drug is inactivated in the donor phase [38]. 

Terauchi et al. prepared an MLT/HP-*β*-CD complex in order to enhance MLT’s water solubility and the impact that this complex has on dental tissue regeneration and osteogenic differentiation in MC3T3-E1 cells. In order to prepare the complex, MLT was co-diluted with the CD in a phosphate buffer (7.4) and the mixture was shaken at room temperature. The suspensions were passed through a 0.22 μm filter, the filtrate was diluted with methanol and water, and the solubility of MLT was calculated using HPLC. The stability constant was calculated by conducting phase solubility studies and was found to be 124.6 M^−1^, which is similar to that of the MLT/*β*-CD complexes calculated by other researchers [31,34,37,44]. The complexes were characterized by NMR and XRD, and their effect on osteogenic differentiation was examined. The results showed an increased activity of alkaline phosphatase (ALP), the formation of mineralized matrix, and the expression of osteogenic differentiation genes, all of which indicate that the cell uptake of MLT from the MLT/CD complex was greater than the uptake of plain MLT [39]. 

Moreover, injectable MLT/CD complexes have also been investigated. Johns et al. studied formulations for intravenous administration of MLT. Two different formulations were tested for their stability, pharmacokinetics and pharmacodynamics. Both formulations contained 5 mg of MLT, propylene glycol (PG), HP-*β*-CD and phosphate buffer (pH = 7.0). One of the formulations contained sodium bisulfite as an antioxidant and NaEDTA as a chelating agent. Preliminary studies suggested that the best ratio between PG and HP-*β*-CD was 10 and 20%, respectively. The solution that contained the antioxidant and the chelating agent was more stable over a period of 31 days, with changes in pH and the solution’s color being more subtle than those of the solution that did not [40]. In another study, a solution of D-*β*-hydroxybutyrate (BHB), MLT and CD was prepared in order to treat hemorrhagic shock. MLT was dissolved in a mixture of 10% HP-*β*-CD, 5% polyvinylpyrrolidone (PVP) and 5% polyethylene glycol (PEG). Equal parts of this MLT solution and 4 M BHB (pH = 7.4) were prepared to form the final solution, which was filtered through a 0.2 μm filter paper and lyophilized. The PVP, like other water-soluble polymers, increased the stability of the complex. The prelyophilization solution (frozen solution) and the lyophile were characterized using DSC, while the lyophile was also characterized by XRD. The DSC revealed that in the prelyophilized solution, PVP and HP-*β*-CD, which are both non-crystallizing, inhibited the crystallization of BHB. In the final lyophile, BHB was crystalline, indicating that the crystallization occurred during the drying stage of crystallization. The BHB/MLT/CD lyophile gave a broad endotherm from 80 to 120 °C, which may be attributed to the BHB dehydration and water vaporization. The XRD studies indicated, as mentioned before, that BHB crystallized (BHB•0.25 H_2_O) and that the MLT/CD lyophile is amorphous [41]. 

Transdermal delivery of MLT using MLT/CDs complexes has also been studied. In the research work conducted by Lee et al., the percutaneous absorption of MLT was investigated. Solutions of MLT in buffer (pH = 6.1), 20% PG solution, 40% PG solution, and 40% PG plus 30% HP-*β*-CD were used. From their previous study [48], it was shown that MLT had the best solubility in the mixture of 40% PG and 30% HP-*β*-CD. Regarding diffusion studies, Franz type diffusion cells were used with MLT solutions applied to the donor’s chamber and 0.9% physiological saline (pH = 5.5) to the receptor phase. MLT showed increased permeability only when hairless mouse skin (HMS) membrane was used. This increase is believed to be a result of the increased solubility of MLT and of the ability of cyclodextrins to disrupt the lipid membranes. When ethylene vinyl acetate (EVA) membranes were used, the permeability decreased when solubility increased; finally, when microporous polyethylene (MPE) membranes were used, MLT showed very low or negligible diffusion [42]. In addition, a modified release reservoir type transdermal delivery for MLT was developed using 40% PG in a phosphate buffer (pH = 6.1) or 40% and 30% HP-*β*-CD in a phosphate buffer. The highest permeability was achieved from the EVA membrane that contained 28%VAc. MLT showed better solubility when PG and HP-*β*-CD were used instead of just PG. However, the permeability was not better even though the increase in solubility implied an increase in permeability [43].

In order to study the nature of MLT/CD complexes, many researchers have produced liquid forms and examined their properties. Bongiorno et al. studied the stoichiometry, geometry, stability, and solubility of complexes of MLT with *α-*, *β-*, and *γ*-CD with the use of NMR spectroscopy, mass spectrometry, solubility measurements and calorimetric measurements. For the NMR studies, MLT and cyclodextrins were suspended in D_2_O and stirred at room temperature, and the insoluble MLT was then filtered. The results from the NMR experiments indicated that the plot complex of MLT/*α*-CD corresponded to 1:1 stoichiometry, whereas that of the *β-* and *γ*-CD did not correspond to either 1:1 or 2:1. This might be a result of the multiple equilibria in the solution. For the mass spectrometry studies, solutions of MLT with the different CDs in a mixture of acetonitrile and water (1:1) were mixed in order to form a 1:1:1:4 mixture of *α*-CD, *β*-CD, *γ*-CD and MLT. The results showed that the order of stability was *β*-CD > *γ*-CD > *α*-CD. For the solubility tests, aqueous solutions of MLT and different concentrations of the various CDs were used. The results indicated that the stoichiometry of the complexes was 1:1 and that the opalescence of the *γ*-CD solution at higher concentrations of the CD was a result of the formation of MLT/CD aggregates. Finally, for the calorimetric experiments, the calorimetric cell was mixed with water and the CD to give the signal used as a baseline, and then water was replaced with an aqueous solution of MLT (0.002 mol/kg). The results from solubility experiments agreed with the fact that at low CD concentrations, the stoichiometry of the complex formed is 1:1, while for increased concentrations it could be of a higher order or even form aggregates. From the calorimetric experiments, it is clear that the *β*-CD formed the most stable complex with an association constant *K*_b_ = 48.0 ± 0.6 M^−1^, while the small cavity of *α*-CD did not permit full insertion of the MLT, leading to the most unstable complex. Regarding the *a*-CD/MLT complex, the spectrum showed that there were NOEs of the H-3′ with H-6, H-4 and H-7 of MLT, but not with H-2, and a cross peak between the H-6-H-5′, leading to the conclusion that MLT C-4, C-5 and C-6 are inserted into the CD cavity and that MLT possibly approaches from the wider rim. For the *β*-CD/MLT, the ROE data indicate that the H-3′ and H-5′ of the *β*-CD interact with the H-4 and H-7 and that there is also a dipolar interaction between H-3′ and H-2. Compared to the *α*-CD/MLT complex, MLT is more deeply inserted into the CD cavity, entering the cavity from the larger rim with H-6 being close to H-6′ and H-5′ but far away from the H-3′, and H-2 being close to H-3′ but not to H-5’. The *γ*-CD ROE data did not give any specific information about the host–guest interactions. All of the aromatic protons interact with the H-3′ and H-5′, which indicates the full insertion of MLT into the *γ*-CD cavity, but the depth of insertion and specific interactions could not be elucidated. In the mass spectroscopy (MS) experiments, the order of stability between *α-* and *γ*-CD was reversed, possibly due to the fact that the gas phase probably adds stability to the MLT/*α*-CD complex [44].

Another group of researchers studied the ability of MLT to form complexes with *β*-CD at a variety of pH values (1, 3, 5, 7, 11.5), as well as their stability. The complex of *β*-CD in an aqueous solution, when protected from oxygen and light, showed satisfactory stability over a wide range of pHs. The inclusion constants of MLT were estimated by UV-Vis spectrophotometry at 278 nm for pH = 3, 7 and 11.5, and ranged from 2.94 ± 0.01 M^−1^ to 3.07 ± 0.06 M^−1^. For pH = 3, the constant was also calculated electrochemically by a voltametric study and was found to be 3.15 ± 0.01 M^−1^, which is very close to the one found spectrophotometrically (3.07 ± 0.06 M^−1^) [45]. Additionally, in an effort to improve MLT’s solubility, Lee studied the solubility and stability of MLT after its solubilization in propylene glycol (PG) and incorporation into 2-HP-*β*-CD. Different concentrations and combinations of PG and 2-HP-*β*-CD were used. Using the PG solution, the solubility of MLT increased slowly up to 40% PG concentration, and then rose steeply. Keeping the PG concentration constant and increasing the 2-HP-*β*-CD concentration, MLT’s solubility increased linearly. The most efficient combination where MLT showed increased solubility was 40% PG and 30% 2-HP-*β*-CD. The solubility studies were conducted at pH = 1.4 and 7.4, in order to simulate the gastric and intestinal fluids, respectively. The stability of MLT was studied under different pH values (1.4, 4.7, 7.4, 10), at 70 °C. MLT was unstable at pH = 1.4, but stable at the other values. In the solutions that contained only PG, MLT stability decreased drastically with slight increases in the PG concentration and increased again at higher PG concentrations, while in solutions that contained PG and 2-HP-*β*-CD, the stability of MLT was close to that in the aqueous solution [48]. Scientists have also focused on the effect that a solvent (MeOH, *n*-PrOH, acetonitrile (ACN)) can have on the fluorescence of some substances, including MLT and their complexes with *β*-CD and HP-*β*-CD, as well as their stability. The study was conducted using a phosphate buffer (pH = 6.994). MLT in water was diluted with 95% buffer and water or 68% buffer and a co-solvent (ACN, *n*-PrOH or MeOH). The complexes of the MLT became weaker and destabilized at increasing concentrations of solvent, due to the fact that the solvent competes against the hormone for the CD cavity. For MLT specifically, the spectrofluorimetric method showed that the highest formation constant (*K*_AP_ = 106 ± 2 M^−1^) was achieved with 1% ACN [49]. 

In an attempt to increase the efficacy of drug delivery systems, researchers have also investigated computationally the possible interactions of MLT with CDs. The study started with conformational studies of MLT, as well as an investigation of the ability of MLT to form a dimer. The possible interactions for the formation of a dimer were head–head, head–tail, tail–tail and indole–indole, where the head of MLT is the upper part that contains the methoxy and amide groups and the tail is the lower part that contains the indolic NH. In order to identify which MLT conformation was more accurate, the results from FT-IR computations were compared to the results from FT-IR of the solid state. From the peaks of the carbonyl, the methoxy group, the double bonds and the Ar-O bond, it was concluded that the best conformation was the one achieved with CRY/PBEh-3c. The lowest energy conformation of the CD was then investigated, followed by an examination of the electrostatic host–guest interactions of the MLT/CD complex with the use MOLDRAW. The most stable structure was found by the xTB-GFN2, and the data from the FT-IR showed good accordance with the solid-state results, taking into account the accuracy and computational cost. The molecular dynamics studies provided a realistic inclusion process, not only in the gas phase but also in water and acetonitrile. In the future, studies will be extended from the gas phase to water so that the solvation effects, as well as the use of substituted CDs, can be further analyzed [50]. 

## 4. Methods

Several approaches have been taken into consideration to ensure a high-quality literature review of MLT/CD inclusion complexes. Two main databases (Science Direct and Google Scholar) were used, applying the main keywords: melatonin, cyclodextrins and inclusion complexes. In particular, the search performed in September 2021 with an adjusted time frame of 1990 to 2021 yielded 2040 results in Google Scholar and 884 results in Science Direct. Only suitable research articles were selected from this set, while duplicates were removed. 

## 5. Conclusions

The pineal hormone melatonin has a major role in the regulation of many functions of the human body and has a positive effect in the treatment of various diseases, mainly sleeping-related disorders. MLT’s partial water solubility leads to an insufficient absorption; additionally, in combination with the extended first pass effect, the levels of MLT in the plasma do vary from person to person. Inclusion of drugs into CDs is a way of increasing the solubility of many poorly water-soluble drugs, such as melatonin. MLT’s inclusion leads to a 1:1 complex with the majority of CDs, resulting in increased solubility of the hormone. The interactions of MLT with CDs have been studied using a variety of techniques, including NMR, FT-IR, XRD and DSC. In vivo experiments showed an increase in the uptake of MLT upon inclusion into CDs. For solid and semi-solid complexes of MLT/CD, *β*-CD (or a modified *β*-CD) was preferred. However, studies have also been conducted with other CDs. For the liquid forms, again, *β*-CD (or a modified *β*-CD) was preferred, with a limited number of studies for the other CDs. The interactions between MLT and the CD are critical for the successful inclusion of the drug, so the size of the cavity is a crucial parameter for the choice of host. The tendency toward usage of *β*-CD analogues is probably due to the fact that the size of the cavity in these CDs is ideal for hosting MLT. Studies conducted with other CDs proved that for the purpose of hosting MLT, *α*-CD had a relatively small cavity, whereas that of *γ*-CD was considerably larger.

## Figures and Tables

**Table 1:** Summary table of published experimental studies on MLT/CD inclusion complexes.

Dosage Form *	Release Rate *	Purpose of Study	CD Used	Method of Inclusion	Studies Conducted	pH of Conducted Experiments	Reference
solid	immediate	effect on osteosarcoma MG-63 cell lines, increase solubility	HP-*β*-CD	MW of solution	phase solubility, FT-IR, DSC, TGA, NMR, SEM, XRD, drug release	7.4 (drug release)	[27]
	immediate and slow	osteoinductive and anticarcinogenic properties for osteosarcoma therapy	HP-*β*-CD	MW of solution	SEM, ATR-FT-IR, drug release	7.4 (drug release)	[28]
	sustained	antitumor activity on osteosarcoma cell lines	*β*-CD		FT-IR, drug release	5.2–5.3 (drug release), 7.4 (drug loading, release)	[29]
	N/A	improve MLT hydrophilicity	RM-*β*-CD	co-evaporation	phase solubility, DSC, FT-IR, fluorescence, UV-Vis spectroscopy	7.5 (stability)	[30]
		properties of inclusion complexes	α-CD, *β*-CD, *γ*-CD, HP-*α*-CD, HP-*β*-CD, HP-*γ*-CD, SBE-*β*-CD, mono-G_2_-*β*-CD, Me-*β*-CD	physical mixing, kneading, freeze-drying	phase solubility, NMR, DSC, XRD	4, 7.5, 11 (solubility)	[31]
		formation and stability of inclusion complexes	*α*-CD, *β*-CD, *γ*-CD,	physical mixing, lyophilized and crystalline complexes	FT-IR		[32]
	fast and sustained	modified release bilayered tablet	*β*-CD	solid dispersion, kneading	phase solubility, drug release, stability	1.2, 6.8 (drug release)	[33]
	controlled	tablets for problems of sleep onset/maintenance	*α*-CD, *β*-CD, *γ*-CD, HP-*β*-CD, sulfated-*β*-CD, HP-*α*-CD and HP-*γ*-CD	physical mixing, co-evaporation	UV-Vis, NMR, drug release	1.2 and 7.4 (drug release)	[34]
		transdermal delivery cotton fibers	*β*-CD	lyophilization	ζ-potential, SEM, elemental analysis, XRD, DSC, ATR-FT-IR	7.4 (drug release)	[35]
semi-solid	controlled	in vitro release of MLT from hydrogels	*β*-CD	simple mixing	phase solubility, hydrogels swelling, drug release	1.4–7.4 (stability)	[36]
liquid	immediate	solution/suspension for nasal delivery	HP-*β*-CD, RM-*β*-CD	flash evaporation (co-evaporation)	phase solubility, DSC, HPLC, permeability		[37]
		eye solution for granular dystrophy type 2	*α*-CD, *β*-CD, *γ*-CD, HP-*β*-CD	solution of MLT, CD and PBS	phase solubility (all CDs), liquid phase analysis, in vitro permeation, *in vivo*, stability (HP- *β* -CD)	7.4 (inclusion), 6.0, 6.8, 7.4, 8.0 (stability)	[38]
		enhance cellular uptake and dental tissue regeneration	HP-*β*-CD	shake bath treatment of solution	phase solubility, NMR, XRD, DFT, cellular uptake and intracellular trafficking, GC-MS	7.4 (solubility)	[39]
		IV formulation	HP-*β*-CD	simple mixing	solubility (clarity), stability, sterility	7.0 (solubility)	[40]
		IV solution for hemorrhagic shock treatment	HP-*β*-CD	simple mixing	DSC, XRD	7.4 (inclusion)	[41]
		solution for transdermal delivery	HP-*β*-CD	water bath treatment of solution	phase solubility, diffusion	6.1 (inclusion), 5.5 (diffusion)	[42]
	modified	transdermal delivery (reservoir type)	HP-*β*-CD	simple mixing	membrane, adhesion, device	6.1 (inclusion)	[43]
	N/A	stoichiometry, geometry, stability and solubility of complexes	*α*-CD, *β*-CD, *γ*-CD	stirring or ultrasonication or thermostatic bath treatment of solutions	phase solubility, NMR, DSC, MS		[44]
		stability, estimation of pKa and value of inclusion	*β*-CD	simple mixing	stability, UV-Vis, voltammetry	1, 3, 5, 7 and 11.5 (inclusion and stability)	[45]
		thermal behavior, purity, solubility and stability	HP-*β*-CD	water bath treatment of solutions	solubility, stability	1.4, 7.4 (solubility, stability), 6.1 (solubility) and 10 (stability)	[46]
		alternative solution to organic solvents in molecular fluorescence	HP-*β*-CD, *β*-CD	simple mixing	stability	~7 (inclusion)	[47]

* Dosage form and release rate, as stated by the author(s). ATR-FT-IR: attenuated total reflectance Fourier transform infra-red, BHB: *β*-hydroxybutyric acid, CD: cyclodextrin, CDI: carbonyldiimidazole, DFT: density functional theory, DSC: differential scanning calorimetry, FT-IR: Fourier transform infrared, G_2_: 6-*O*-*α*-maltosyl, GC: gas chromatography, GMA-EDA: glycidyl methacrylate-ethylenediamine, HEA: 2-hydroxyethyl acrylate, HP: hydroxypropyl, HPLC: high performance liquid chromatography, IV: intravenous, Me: methyl, MS: mass spectrometry, MW: microwave, N/A: not stated, NMR: nuclear magnetic resonance, NS: nanosponge, PBS: phosphate-buffered saline, PEG: polyethylene glycol, PG: propylene glycol, PVP: polyvinylpyrrolidone, RM: random methylated, SBE: sulfobutylether, SEM: scanning electron microscope, TGA: thermal gravimetric analysis, UV-Vis: ultraviolet-visible, XRD: X-ray diffraction.

## Data Availability

Not applicable.
